# Disgust and Contamination: A Cross-National Comparison of Ghana and the United States

**DOI:** 10.3389/fpsyg.2013.00091

**Published:** 2013-02-27

**Authors:** Alexander J. Skolnick, Vivian A. Dzokoto

**Affiliations:** ^1^Department of Psychology, Saint Joseph’s UniversityPhiladelphia, PA, USA; ^2^Department of African American Studies, Virginia Commonwealth UniversityRichmond, VA, USA

**Keywords:** disgust, contamination, disease susceptibility, behavioral immune system, Ghana, West Africa, moral disgust

## Abstract

The emotion of disgust, with feelings of revulsion and behavioral withdrawal, make it a prime emotion to aid in the avoidance of sources of contamination, including sources of potential infectious disease. We tested the theory that living in a region with a historically high prevalence of infectious diseases would promote higher levels of disgust and contamination sensitivity as a protective measure. A sample of undergraduates from Ghana (*n* = 103, 57 women), a country with a historically high prevalence of infectious diseases, showed significantly higher scores on scales assessing disgust, contamination, and disease susceptibility than a sample of undergraduates from the United States (*n* = 96, 58 women), a country with lower levels of disease threat. Contamination sensitivity mediated the national differences in disgust. Disgust connoting contamination also produced larger cross-national effect sizes than other types of disgust. Finally, a factor analysis on the Ghanaian responses to one of the disgust scales did not resemble the usual three-factor solution found in West. Taken together, the results were consistent with the hypothesis that a region with a higher prevalence of infectious disease threats would produce greater sensitivity to disgust and contamination than seen in lower disease threat regions. This first study on disgust in Africa showed that disgust sensitivity could differ considerably from that in the West.

## Introduction

Disgust is considered a “basic” emotion, with demonstrated evidence for universality in disgust-associated physiology, expressions, and antecedents (Ekman, 1992; Rozin et al., 2008). While the experience of disgust is likely found worldwide, what is considered disgust-evoking are in some cases universal (e.g., feces, incest) and other cases culture-specific (e.g., eating insects or blood). From the first modern studies of disgust, contamination themes have been central (Rozin and Fallon, 1987). Curtis and Biran (2001) argued that there are common categories of disgust-evocation across societies and these mainly encompass aspects of contamination and contagion. Disgust-related cues may both warn one to the presence of contaminating stimuli as well as promote behavioral avoidance away from such stimuli. Davey (1994) and Matchett and Davey (1991) made the early argument that disgust of certain animals could best be explained by a disease avoidance function of disgust. Moreover, Oaten et al. (2009) make the extensive argument that a disease avoidance function for disgust fits with most of the types of elicitors of disgust, not just certain animals.

In recent years, there has been accumulating empirical evidence for an association between disgust sensitivity, contamination, and disease avoidance. For example, Curtis et al. (2004) showed that pictures connoting contamination were rated by a global sample as more “disgusting” than similar non-contamination-related control pictures. Several studies, using different populations (e.g., Italians, Germans, European Americans, African Americans), have found significant positive correlations between various disgust scales and scales that measure contamination fears (Sawchuk et al., 2000; Mancini et al., 2001; Schienle et al., 2003; Olatunji et al., 2004; Williams et al., 2012). Furthermore, Deacon and Olatunji (2007) extended this finding by showing that disgust sensitivity predicts both emotional and behavioral responses (e.g., behavioral avoidance) to contamination-related stimuli. This relationship held even when controlling for possible confounding variables such as anxiety and depression levels. In sum, there is growing support for the association between disgust and avoidance of contamination and disease.

If avoidance of contamination is a determining feature of disgust elicitation (Schaller and Duncan, 2007; Oaten et al., 2009), then we might expect that sensitivity to disgust would be higher in places where risk of contamination or disease is also elevated. In fact, Oaten et al. (2009) hypothesized that global variation in pathogen prevalence may predict cross-national differences in disgust sensitivity. For example, in more tropical regions where there are greater threats of infectious diseases we might expect higher disgust sensitivity to potential cues of a contaminating substance. This hypothesis converges with Schaller’s (2011) argument that people act defensively against perceived threats of contagious diseases as part of a “behavioral immune system,” and disgust is the emotional reaction helping to promote avoidance of such threats. In this vein, one would expect that in areas of the world where contagious disease is more threatening, greater sensitivity to disgust could develop to avoid potential mortal threats. In contrast, Oaten et al. (2009) also suggested a population with a history of encountering sick individuals and poor environmental conditions that foster disease threats (e.g., poor waste management) and inadequate hygiene may instead lead to lower disgust sensitivity due to repeated exposure effects. Despite these two perspectives making different predictions, this question has not been empirically tested. No study to date has compared disgust sensitivity between Western countries with lower disease threats to non-Western countries with high disease threats.

The present study is the first to empirically examine a potential association between historical prevalence of infectious pathogens and levels of disgust. West African countries received the highest ratings in the world on the index of disease prevalence (IDP) generated by Murray and Schaller (2010). By examining disgust in Ghana, a country with one of the highest ratings for historical infectious disease prevalence (Ghana = 1.16; IDP highest = 1.17), we can test the hypothesis that prevalence of infectious pathogens is linked to levels of disgust sensitivity. In particular, we hypothesized that Ghanaians would score higher in disgust and contamination sensitivity than people from countries with lower ratings for their history of disease prevalence, such as from the United States (−0.89; IDP lowest = −1.31). Given the potential for greater threat of infectious diseases, we should expect that forms of disgust sensitivity related to contamination would be most protective (or adaptive) in a country such as Ghana. To further elucidate this link between disease threat and sensitivity to contaminating agents, we examined a mediation model for whether differences in disgust sensitivity levels between Ghana and the U.S. could be explained by higher levels of contamination sensitivity in the Ghana sample (as measured by the contamination subscale of the Padua Inventory, Sanavio, 1988).

In addition to potential differences in contamination sensitivity, aspects of West African culture suggest that disgust itself may be different in Ghana compared to the U.S. One is that Ghanaians are described as strongly collectivist and likely to consider negative emotions (e.g., anger, fear, sadness) undesirable (Kim-Prieto and Eid, 2004), and, thus, less likely to express them (Matsumoto et al., 2008). Additionally, Dzokoto (2010) found Ghanaians paid less attention to their emotions overall than Americans but more attention to their bodily activations. As such, we considered whether disgust sensitivity is structurally different in the Ghanaian sample compared to a U.S. sample, given the cultural difference of greater focus on the body and visceral reactions. Olatunji et al. (2007, 2009) has shown the standard disgust sensitivity scale, the Disgust Scale Revised (DS-R; Haidt et al., 1994) divides into three factor-derived subscales that match Rozin et al.’s (2008) divisions of disgust based on theoretical considerations: core disgust (12 items), animal reminder disgust (8 items), and contamination disgust (5 items). This three-factor solution seems robust as it was found in samples from seven countries besides the U.S., including four European nations, Australia, Brazil, and Japan (Olatunji et al., 2009). Core disgust sensitivity is based on a sense of offensiveness and oral incorporation (e.g., rotting meat, vomit, mucus). Animal reminder disgust sensitivity is related to stimuli that may remind humans of their animal origins (e.g., death and injuries that expose the innards of the body). Contamination disgust sensitivity is related to contaminating agents (e.g., toilet seats), contagion (e.g., cook with a cold), and sympathetic magic that involves stimuli that resemble such agents, such as chocolate in the shape of dog feces. Given the higher potential of disease threats in West Africa, we hypothesized that contamination disgust sensitivity would be higher in Ghana than in the United States, whereas we had no specific expectations for core disgust or animal reminder disgust to vary by country. To test these predictions, we compared disgust sensitivity in Ghana to the U.S. based on the standard three-factor model. We further assessed the factor structure of disgust sensitivity in Ghana to test whether Ghanaians produced factors that resemble core, animal reminder, and contamination disgust.

In sum, we had several goals examining disgust and contamination sensitivity in making cross-national comparisons between Ghana and the United States. First, we were interested in whether Ghanaians had higher disgust and contamination sensitivity than Americans, as predicted by the theory that disgust sensitivity is related to the historical prevalence of disease and contamination in a society. If higher sensitivity to disgust results in avoidance of contaminating agents, then a historical legacy of infectious diseases should heighten disgust sensitivity and disgust should be strongly related to cues that connote contamination. Therefore our second goal was to examine whether disgust was linked to contamination to a greater extent in Ghana compared to the U.S. We examined this goal in two ways. First we tested whether contamination sensitivity mediated the potential cross-national differences in disgust sensitivity. We also tested specific predictions that disgust subscales related to contamination (e.g., contamination disgust from the DS-R, pathogen disgust from the TDDS) were likely to differ cross-nationally to a greater extent than disgust less related to contamination (e.g., animal reminder disgust from the DS-R, moral disgust from the TDDS). Third, we wanted to see if Ghanaians’ concept of disgust was similar to or different than the American construct of disgust.

## Materials and Methods

### Participants

The present sample of participants consisted of 103 undergraduates (57 women) from the University of Ghana and 96 undergraduates (58 women) from Saint Joseph’s University, in Philadelphia, PA, USA. The mean age of the Ghanaian college students was 25.3 years (SD = 4.50, range 20–50). Of the 22 different ethnic groups in the Ghanaian sample, the largest were the Akan (33%), Ga (20%), and Ewe (17%). The sample self-identified as non-Catholic Christian (89%), Muslim (9%), and Catholic (2%). The American sample of undergraduate students had a mean age of 19.6 years (SD = 1.10, range 18–22, seven students did not report their age). The students were 84.4% White European American, 6.4% East Asian, 5.2% Latino, 2% African American, and 2% other. Religious affiliation was not queried at Saint Joseph’s University, but the large majority of the campus population is Catholic.

### Materials

All scales used in Ghana were converted to British spelling and some words were modified to better represent Ghanaian knowledge and increase understanding.

#### Disgust scale revised

Disgust sensitivity was assessed with the 25-item version of the DS-R, with a 5-point scale (0–4) for rating agreement level with statements about being bothered by disgusting events (e.g., *If I see someone vomit, it makes me sick to my stomach*) and for rating how disgusting certain experiences are (e.g., *You see maggots on a piece of meat in an outdoor garbage pail*; Haidt et al., 1994; modified by Olatunji et al., 2007). Because drinking milk is rare in Ghana, one item, *“You are about to drink a glass of milk when you smell that it is spoiled”* was changed to “…*glass of orange juice*…” In the Ghana sample, Cronbach’s α was acceptable for the overall disgust sensitivity score (0.69) and for two of the three subscales: α = 0.73 for core disgust, α = 0.77 for animal reminder disgust, and α = 0.56 for contamination disgust. The Cronbach’s α values were similar in the American sample with α = 0.91 for overall DS-R score, 0.83 for core disgust, 0.84 for animal reminder disgust, and 0.57 for contamination disgust. The alpha values found in the present sample are typical for this scale.

#### Three domain disgust scale

The TDDS measures disgust sensitivity for pathogen, sexual, and moral domains (Tybur et al., 2009). Only the pathogen and moral subscales were tested as the sex subscale items were dropped due to cultural decorum. Therefore most analyses used the subscales and not the total TDDS mean. Participants were asked to rate each of 14 actions from *not at all disgusting* (0) to *extremely disgusting* (6). Both pathogen disgust (e.g., *stepping on dog poop*) and moral disgust (e.g., *stealing from a neighbor*) were made-up of seven actions. Cronbach’s α was acceptable for the overall disgust sensitivity score (0.85) and for the two subscales: α = 0.75 for pathogen disgust, and α = 0.91 for moral disgust. Similar alphas were obtained in the U.S. sample: α = 0.91 for overall TDDS score, α = 0.87 for pathogen disgust, and α = 0.94 for moral disgust.

#### Contamination subscale of Padua inventory

The Padua is a 10-item subscale of the Padua Inventory that measures distress due to contaminating situations (e.g., *I find it difficult to touch garbage or dirty things*; Padua; Sanavio, 1988). Items are scored on a 5-point Likert-type scale ranging from 0 = *Not at all* to 4 = *Extremely*. The Padua demonstrated acceptable internal consistency in both the Ghanaian sample, α = 0.74, and the U.S. sample, α = 0.87.

#### Perceived vulnerability to disease

The Perceived vulnerability to disease (PVD) measures individual differences in concerns about the transmission of infectious diseases (Duncan et al., 2009). The 15-item PVD produces two subscales: perceived infectability (PI; susceptibility to disease, e.g., *If an illness is “going around,” I will get it*.) and Germ Aversion (GA; emotional discomfort in certain contexts, e.g., *My hands do not feel dirty after touching money*) and assessed mean ratings of feelings from 1 (*strongly disagree*) to 7 (*strongly agree*). The PI subscale had acceptable internal consistency, α = 0.71, but the GA subscale had poor internal consistency, α = 0.39. This lack of consistency is likely due to several items on the Germ Aversion subscale not relating well to Ghanaian culture. For example, coming into close contact over things like sharing food, shaking hands, and visiting sick people are normative behaviors, and students might avoid used clothes for reasons of status, not contamination. The U.S. sample showed acceptable values: α = 0.84 for the PI subscale and α = 0.71 for the GA subscale.

#### Religiosity scale

The Religiosity scale has six items that address religious and spiritual feelings, as well as commitment to religious teachings and practice (Cohen et al., 2006). All items were rated on 0 (*Not at all*) to 5 (*Extremely*) scales. Alpha values were good in both samples, α = 0.92 in Ghana and α = 0.93 in the U.S.

### Procedure

The study was approved by Saint Joseph’s University’s Institutional Review Board. Ghanaian participants read and signed an informed consent form. They then filled-out a brief demographic sheet (gender, age, ethnicity, religion), and a packet of the surveys randomly ordered: religiosity scale, DS-R, TDDS, Padua, and the PVD. They participated in groups of about 15–20 in a classroom setting. Ghanaian participants were compensated with cell phone vouchers. The U.S. students completed the surveys online after reading the consent form. The U.S. students were compensated with partial class credit for their participation.

## Results

### Preliminary results

When comparing the African and American samples, no differences were seen across ethnic or religious affiliations for any variable of interest. However, two differences emerged: the Ghanaian students (*M* = 3.74, SD = 0.82) scored significantly higher on the religiosity scale than U.S. students (*M* = 2.73, SD = 1.06), *t*(179.15) = 7.47, *p* < 0.001, *d* = 1.07; and Ghanaian students were significantly older than U.S. students, *t*(115.73) = 12.27, *p* < 0.001, *d* = 1.71. Both of these variables correlated with disgust and contamination scores: highest significant *r*-value for religiosity was with mean DS-R, *r*(196) = 0.142, *p* = 0.046; highest significant *r*-value for age differences was with mean DS-R, *r*(189) = 0.162, *p* = 0.025. To check whether religiosity levels or age predicted disgust and contamination scores beyond that predicted by country differences, a series of hierarchical regressions were run entering either mean religiosity or age first and then country. In all cases where disgust and contamination factors differed by country (see below and Table 1), religiosity or age were significant predictors in the model. However, when country was added to each regression, country was the sole significant predictor of the disgust or contamination measure, except for the Padua scores, where mean religiosity and age remained significant predictors of Padua scores along with the country factor. Thus, although the two samples differed on religiosity and age, these variables were not important predictors of the disgust and contamination measures.

**Table 1:** **Cross-national comparisons between mean responses to the disgust and contamination surveys**.

Survey	Ghana		USA		*p*	*d*
	Mean	SD	Mean	SD	
DS-R (mean)	2.52	0.52	2.22	0.69	0.001	0.49
DS-R core	2.60	0.57	2.46	0.72	0.140	0.22
DS-R animal reminder	2.52	0.67	2.34	0.94	0.135	0.22
DS-R contamination	2.35	0.73	1.43	0.70	<0.001	1.29
TDDS (mean)	4.18	1.09	3.27	1.25	<0.001	0.78
TDDS-pathogen	4.48	1.07	3.65	1.24	<0.001	0.72
TDDS-morality	3.88	1.64	2.89	1.70	<0.001	0.59
Padua	1.64	0.63	1.02	0.72	<0.001	0.92
PVD-PI	3.38	1.23	3.28	1.38	0.687	0.06
PVD-GA	4.17	0.91	3.19	1.13	<0.001	0.97

**p*, *p*-values for between-country independent *t*-tests run on each mean score (Ghana *n* = 101–102; U.S. *n* = 96); *d*, Cohen’s *d* measure for between-country differences in effect size; DS-R, Disgust Scale Revised; TDDS, three domain disgust scale; Padua, contamination subscale of the Padua inventory; PVD, perceived vulnerability to disease scale; PI, perceived infectability subscale; GA, germ aversion subscale*.

### Cross-national comparisons of disgust and contamination

We tested country differences for all the scales with independent *t*-tests and used corrected degrees of freedom if the Levene’s test was significant. Cohen’s *d* was calculated for effect sizes for all country comparisons. Table 1 presents the mean scores for the disgust and contamination measures used, along with the *p*-values and effect sizes for cross-national comparisons. Assessing whether disgust sensitivity was higher in a society with a history of higher prevalence of infectious disease as compared to a society with a history of lower prevalence of infectious disease, we found that both measures of disgust, mean DS-R scores and the two scales of the TDDS, were significantly higher in Ghana than in the U.S., DS-R: *t*(176.22) = 3.50, *p* = 0.001, *d* = 0.49; TDDS-pathogen: *t*(196) = 5.02, *p* < 0.001, *d* = 0.72; TDDS-moral: *t*(196) = 4.17, *p* < 0.001, *d* = 0.59. Scales that addressed feelings about contamination showed similar but larger differences between countries. Mean Padua scores were significantly higher in Ghana than in the U.S., *t*(196) = 6.41, *p* < 0.001, *d* = 0.92 (Table 1). Analyzing the two subscales of the PVD showed that Ghanaians scored significantly higher than the U.S. sample on the subscale that addressed avoidance of germs (PVD-GA), *t*(181.56) = 4.78, *p* < 0.001, *d* = 0.97, but no difference was seen on the scale that addressed one’s perceptions of infectability (PVD-PI), *t* < 1 (Table 1). The much larger effect for the PVD-GA than the PVD-PI was consistent with Ghanaians showing elevated contamination responses, and consistent with Duncan et al. (2009) who found only mild correlations between PI and disgust measures. Thus, mean levels of sensitivity to disgust and contamination were significantly higher among students from Ghana than students from the U.S. supporting our first hypothesis that a country with a history of contagious disease threats would be higher in disgust in comparison to a country that poses less of a threat.

### Mediation analyses

**Figure 1:**
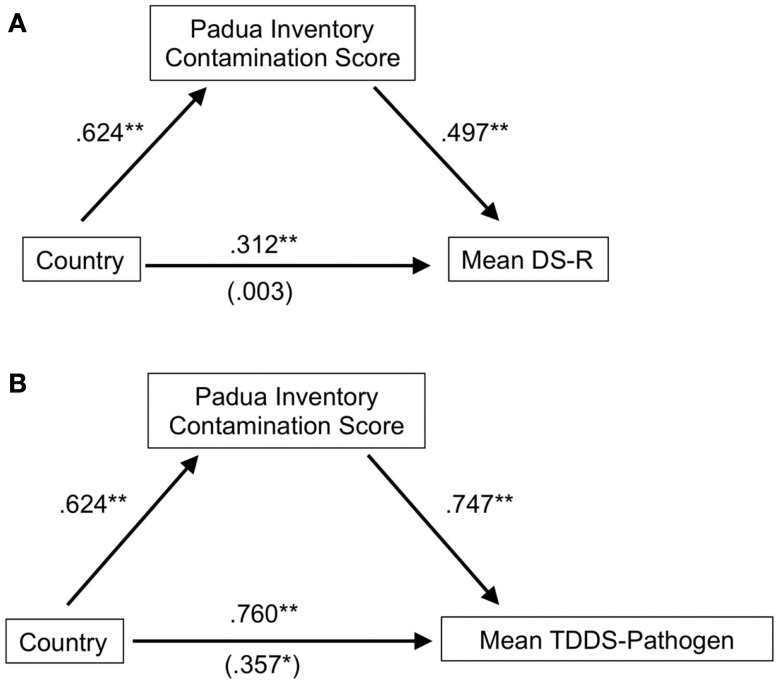
**Results of mediation analyses for the association between country (U.S. = 1, Ghana = 2) and the Disgust Scale Revised (A) and the TDDS-pathogen (B)**. Values equal unstandardized β coefficients. Values in parentheses equal unstandardized β when the mediator was also a predictor of the disgust scale measure.

To further examine the nature of the cross-national differences in disgust and whether contamination concerns might be driving the differences in disgust, we conducted mediation analyses to test whether contamination sensitivity, as measured by the Padua, mediated the differences in disgust evident between Ghana and the U.S. The top portion of Figure 1 depicts the mediation of the association of country (dummy coded as U.S. = 1, Ghana = 2) and disgust sensitivity measured by the DS-R. To meet the criteria for mediation, we first showed, in a series of stepwise regression analyses, a significant relationship between the country and mean DS-R scores, β = 0.312, SE = 0.089, *t* = 3.51, *p* = 0.001. When we added the potential mediator–Padua scores–the relationship between country and the DS-R scores almost disappeared, β = 0.003, SE = 0.081, *t* < 1. A significant Sobel test, *z* = 5.16, *p* < 0.001, confirmed that the strength of the association between the country and disgust sensitivity measure significantly decreased when contamination sensitivity was included in the model. A similar mediation effect was seen for the difference between Ghana and the U.S. for the mean TDDS-pathogen scores (bottom portion of Figure 1). We specifically used the pathogen disgust scale because it represents contamination concerns in the TDDS (Olatunji et al., 2012). In this case the relationship between country and TDDS-pathogen was strong, β = 0.760, SE = 0.164, *t* = 4.48, *p* < 0.001, and when the Padua scores were added to the model, the beta values were halved but remained significant, β = 0.357, SE = 0.172, *t* = 2.07, *p* = 0.04. A significant Sobel test, *z* = 4.18, *p* < 0.001, confirmed that Padua scores mediated this relationship as well. While both the DS-R and TDDS-pathogen scales differed between Ghanaian and American samples, both relationships were mediated by scores on the Padua providing further evidence for the importance of sensitivity to contamination in driving the differences between the disgust sensitivity levels of the two countries.

### Cross-national comparisons of individual factors

**Figure 2:**
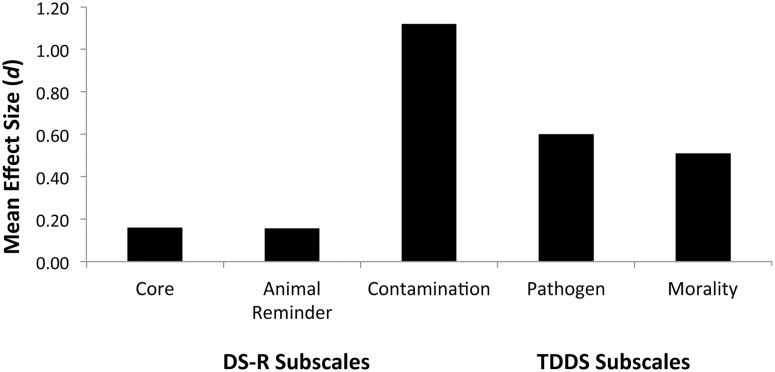
**Mean between-country effect sizes (Cohen’s *d*) for each of the standard subscales of the Disgust Scale Revised and the TDDS**. Positive values indicate Ghanaian disgust sensitivity > American disgust sensitivity.

Previous factor analyses of the DS-R have shown robust cross-cultural evidence for dividing the total DS-R score into three factors: core disgust, animal reminder disgust, and contamination disgust (Olatunji et al., 2007, 2009). Given this standard division, we tested whether Ghanaians would show higher disgust sensitivity to the contamination disgust sensitivity items than Americans. *t*-Tests comparing the DS-R subscales between Ghana and the U.S. found that only the contamination disgust sensitivity scale differed significantly between Ghana and the U.S., *t*(196) = 8.99, *p* < 0.001, *d* = 1.29. Core, *t*(181.59) = 1.49, *p* = 0.140, *d* = 0.22, and animal reminder disgust, *t*(171.31) = 1.52, *p* = 0.135, *d* = 0.22, did not differ greatly between countries. The substantial size of the effect for contamination disgust supports our hypothesis that people living in a country with a history of disease threats would have elevated contamination-related disgust sensitivity. Figure 2, depicting the mean Cohen’s *d* values for each subscale (positive values indicate Ghana > U.S.), highlights the Ghanaians’ robust response to contamination-type disgust items in the scale.

Our prediction that the DS-R subscales related to contamination would differ cross-nationally to a greater extent than disgust less related to contamination was supported. We also predicted that the pathogen subscale of the TDDS, because of its association with contamination-based items, would be found to produce a greater difference between Ghana and the U.S. (Ghana > U.S.) than the moral subscale of the TDDS that is unrelated to contamination. The above analyses showed that Ghanaians produced significantly higher scores on both subscales than those in the U.S. However, the size of the effect for the pathogen subscale (*d* = 0.72) was not substantially larger than the moral subscale effect (*d* = 0.59). Thus, only a trend for an emphasis on contamination when comparing these two measures of disgust sensitivity.

### Factor structure of disgust in Ghana

To examine whether the Ghanaian response to the DS-R overall was similar to the response seen in the U.S., Europe, Japan, and Brazil (Olatunji et al., 2009) we submitted the Ghanaian DS-R data to a factor analysis with varimax rotation and constrained the results to three factors. While the sample size was low for a factor analysis, the Kaiser-Meyer–Olkin measure = 0.650 indicated an adequate sample size. The three factors accounted for 38.60% of the variance (Table 2 shows the factor loading after rotation). Whether using a cutoff criterion of 0.40 (displayed) or higher for the factor loadings, two of the factors contained a mixture of core, animal reminder, and contamination disgust items. The third factor had a majority of animal reminder disgust items, but contained two core disgust items as well. A few scale items had individual KMO values lower than 0.5. Deleting the item with the lowest KMO value (“*Try eating monkey meat*,” 0.346) and rerunning the factor analysis produced a factor structure that accounted for 2% additional variance, but continued to produce factors with a mixture of core, animal reminder, and contamination items that did not resemble the previously established pattern. Thus, the factor structure of the DS-R indicated that Ghanaians responded to the overall scale differently than other Western populations typically do (Olatunji et al., 2007, 2009).

**Table 2:** **Results from a factor analysis with varimax rotation on the Disgust Scale Revised for Ghana**.

Item^a^	Item theme	Factor 1	Factor 2	Factor 3
22. Core	Changing underwear once a week	**0.747**	0.151	−0.165
27. Core	Step on an earthworm barefoot	**0.704**	−0.016	0.160
24. AR	Touch human ashes	**0.654**	0.380	0.034
21. AR	See exposed intestines after an accident	**0.641**	−0.039	0.032
23. CM	Chocolate in the shape of dog feces	**0.635**	**0.406**	−0.067
25. Core	Smell spoiled orange juice	**0.626**	**0.463**	−0.024
19. AR	Pick up a dead pet cat with bare hands	**0.570**	−0.115	0.347
20. Core	Ketchup on ice cream	**0.486**	−0.133	0.010
26. CM	Inflating an unlubricated condom using your mouth	**0.480**	−0.057	0.140
08. Core	Seeing someone vomit	0.161	**0.704**	0.212
17. Core	Smell urine in a tunnel	0.233	**0.630**	−0.077
18. CM	Drink from glass of acquaintance	**0.440**	**0.570**	−0.120
09. CM	Cook has a cold at restaurant	−0.224	**0.521**	0.393
10. AR	Glass eye taken out of socket	0.119	−**0.509**	0.126
03. Core	Hear throat clearing full of mucus	−0.112	**0.478**	0.092
14. AR	Stay in hotel room where man died	0.202	−0.052	**0.678**
07. AR	Touch dead body	−0.028	0.067	**0.656**
11. Core	Rat runs across your path	0.106	0.072	**0.624**
05. AR	Avoid walking through graveyard	−0.033	0.310	**0.576**
02. AR	Preserved human hand in jar	−0.055	0.309	**0.500**
13. Core	Soup stirred with flyswatter	0.173	−0.126	**0.404**
15. Core	Maggots on meat in garbage	0.396	0.237	0.272
06. Core	See cockroach in a house	0.169	0.176	−0.284
01. Core	Try eating monkey meat	0.141	−0.040	0.168
04. CM	Avoid touching public toilet	0.082	−0.043	0.077

*^a^Includes item number and the standard subscale designation. Core, core disgust; AR, animal reminder disgust; CM, contamination disgust*.

*Bold values represent factor scores >0.40*.

Another indication that the sample from Ghana responded to the disgust sensitivity scales differently than they do in the U.S. was the weak relationship between the two disgust sensitivity scales (Table 3), especially between the DS-R core disgust and TDD-Pathogen disgust. These scales have been shown to be strongly correlated in the two studies that have measured disgust sensitivity with both scales (Tybur et al., 2009, Study 4: *r* = 0.92; Olatunji et al., 2012, Study 4: *r* = 0.75). For the Ghanaian sample, the correlations between the DS-R scales and the TDDS scales were all less than or equal to *r* = 0.19, and none reached significantly reliable levels (Table 3). Furthermore, all the correlations between the DS-R scales and the TDDS-Pathogen scale showed significantly smaller *r*-values from the Ghanaian sample than the U.S. sample, Fisher *z* transformations, all *z* ≥ 4.30, all *p* ≤ 0.001. Although the Ghanaian respondents showed moderately high cross-correlations among the subscales of each disgust sensitivity scale as seen in other studies, the patterns of responses differed from Western samples such that weak relations between the DS-R and the TDDS were produced.

**Table 3:** **Pearson correlations among study surveys for Ghana (above the diagonal) and the U.S. (below the diagonal)**.

	1	2	3	4	5	6	7	8	9	10
DS-R total	–	0.89***	0.77***	0.75***	0.09	0.16	0.02	0.47***	−0.06	0.18^+^
DS-R core	0.92***	–	0.47***	0.59***	0.16	0.19^+^	0.09	0.49***	0.04	0.18^+^
DS-R AR	0.90***	0.70***	–	0.37***	−0.09	0.06	−0.15	0.28**	−0.18^+^	0.06
DS-R CM	0.75***	0.57***	0.57***	–	0.14	0.11	0.12	0.35***	0.12	0.20*
TDDS total	0.58***	0.56***	0.45***	0.49***	–	0.70***	0.88***	0.23*	0.29**	0.13
TDDS path	0.74***	0.71***	0.60***	0.61***	0.79***	–	0.28**	0.17^+^	0.26**	0.23*
TDDS-moral	0.30**	0.30**	0.23*	0.27**	0.89***	0.43***	–	0.19^+^	0.22*	0.03
Padua	0.59***	0.60***	0.43***	0.51***	0.39***	0.54***	0.19^+^	–	0.05	0.34**
PVD-PI	0.30**	0.36***	0.19^+^	0.20^+^	0.06	0.11	0.00	0.17	–	0.20*
PVD-GA	0.67***	0.61***	0.54***	0.63***	0.44***	0.60***	0.21*	0.65***	0.32**	–

*DS-R, Disgust Scale Revised; Core, core disgust; AR, animal reminder disgust; CM, contamination disgust; TDDS, three domain disgust scale; Path, pathogen subscale; Moral, morality subscale; Padua, contamination subscale of the Padua inventory; PVD, perceived vulnerability to disease scale; PI, perceived infectability subscale; GA, germ aversion subscale*.

^+^*p* < 0.10; *p < 0.05; **p < 0.01; ***p < 0.001.

## Discussion

As far as we are aware, this was the first study to examine disgust and contamination sensitivity and PVD in a country with a history of relatively high levels of disease threats and in a parallel country with a history of relatively low levels of disease threats. The overall pattern from the Ghanaian respondents was significantly higher disgust and contamination sensitivities than the U.S. respondents, supporting our hypotheses of elevated disgust and contamination sensitivities in a high disease threat country. Ghanaians showed the highest disgust levels, with the largest effect sizes, on the scales that addressed contamination concerns: the contamination disgust subscale, the TDDS-pathogen scale, the Padua measuring contamination sensitivity, and the PVD-GA measuring tendencies of germ avoidance. Such an emphasis on contamination fits with a society that has faced a historical legacy of a prevalence of infectious diseases (Schaller, 2011). In contrast, the three other domains of disgust addressed in this study, core disgust, animal reminder disgust, and moral disgust, are less associated with contamination, and did not differ cross-nationally to the same extent. Likewise, the PI subscale of the PVD was not different between countries, and in the sample from Ghana was not correlated with the DS-R scales and weakly correlated with the TDDS scales (in agreement with Duncan et al., 2009). Duncan et al. concluded that PI was more related to personal health beliefs than to disgust or contamination, which could explain the lack of cross-national differences seen here.

The mediation analyses and the analysis comparing Disgust Scale factors supported the main hypothesis of higher contamination concerns expected in Ghana than in the U.S. Importantly, the measure of contamination sensitivity (Padua) fully mediated the national differences in DS-R scores and partially mediated the national differences in TDDS-pathogen scores. When we calculated the mean effect size for each of the standard DS-R subscales (core, animal reminder, and contamination) compared cross-nationally, the contamination disgust scales produced a substantially larger effect size than the other two subscales. Taken together, the direct cross-national comparisons clearly indicated differences in disgust and contamination. The specific differences in disgust between Ghana and the U.S. were likely driven by concerns over contaminating agents. In other words, individuals from Ghana showed heightened responses to the contamination-related elements of the disgust scales and the contamination scales (Padua, PVD-GA) themselves.

Following this trend, students from Ghana scored higher on both the pathogen and morality disgust scales from the TDDS than students from the U.S. We predicted a larger effect size for the pathogen scale than the morality scale given disease prevalence theory. However, the difference in effect sizes between Ghana and the U.S. for the pathogen and morality scales was only about 0.13 points, less than the size of a small effect. It is worth pointing out that the large cross-national difference for the morality scale may not represent a trend of Ghanaians to rate everything more disgusting. In his multinational study on appraisal dimensions, Scherer (1997a) found that respondents from African countries (only one West African country, Nigeria, was included in the sample) were the most sensitive to the immorality dimension of antecedent appraisals of any of world region. Scherer (1997b) further found that appraisals of immorality were the strongest determinant of disgust among eight possible appraisal dimensions in the African sample. Scherer’s findings suggest an explanation for the unexpected higher scores on moral disgust in Ghana. We found that the Ghanaian students were significantly more religious than the American students, and given the strong ties between religion and morality (Graham and Haidt, 2010), we can suggest that Ghanaian’s religiosity may prime a stronger connection between immorality and moral disgust than seen in the U.S. sample. Thus, a strong association between morality and disgust in African populations has been seen and may have contributed to the difference seen between Ghana and the U.S.

When we examined the factor structure of the Ghanaian responses to the DS-R, the results did not closely resemble the three-factor solution found in the U.S., Australia, several European countries, as well as in Brazil and Japan (Olatunji et al., 2009). Of the three factors, two consisted of combinations of core, animal reminder, and contamination disgust items, and the third was a little more consistent with four of the six items relating to animal reminder disgust (Table 2). This lack of cross-national concordance in factor structure may be due to Ghanaians relating to the specific items differently than the other national samples. What is interesting is that Ghanaians responded to the five contamination disgust items with much higher disgust ratings than the Americans (Figure 2), yet these specific items did not cluster into one factor. The consistency across eight nations that Olatunji et al. (2009) found prompted them to argue that the standard three factors represent “an internally consistent and theoretically distinct universal class of disgust-relevant stimuli” (p. 247). It may be that people living in regions with higher disease threats show shifts in the way they respond to these items and there are exceptions to the suggested universality of these factors. Obviously, research on additional populations in similar high disease threat nations are warranted to further test the universal nature of the three factors.

The disease prevalence hypothesis, that higher infectious disease threats would promote higher levels of disgust and contamination sensitivities (Schaller and Duncan, 2007; Oaten et al., 2009; Schaller, 2011), was supported by the main findings from the present study. Higher disgust and specifically contamination-related disgust fits with Schaller’s (2011) notion that disgust is the motivating emotion of the behavioral immune system that evolved to keep organisms safe from potentially dangerous pathogens contracted from contact with contaminated agents. Oaten et al. (2009) argued that the history of the prevalence of infectious pathogens might not play a strong role. They hypothesized that having extensive contact with sick individuals that have contracted a disease or died from such a disease might generate exposure effects that would, in effect, lower disgust not elevate it. As the current results represent the first set of pertinent data from a high disease prevalent environment, we can say that disgust certainly was not lower in Ghana. If the level of disease prevalence is playing a role, it is not clear whether it is the historical or current prevalence that produced the effects. Further work is needed in West Africa, as well as in other countries outside of Africa that are high in disease threats, to parse disease prevalence’s role in determining disgust and contamination sensitivity levels.

Other aspects of Ghanaian history and culture may provide alternative or parallel hypotheses for higher disgust sensitivity. For example, people in West Africa, Ghanaians included, tend to describe emotions in ways that suggest the importance of the emotion is not the psychological feelings but the physiological feelings (Ameka, 2002; Geurts, 2002; Dzokoto and Okazaki, 2006). Supporting this emotional labeling, Dzokoto (2010) found Ghanaians reported more attention to their somatic feelings and less attention to their emotions and the opposite pattern in a sample from the U.S. As disgust is often associated with strong visceral feelings, it is possible that increased attention to one’s physical sensations coinciding with disgust may result in West Africans reporting greater disgust overall. We do not suggest that disgust is unique emotion in this regard, so we could hypothesize that Ghanaians also feel other emotions, such as fear in a similar manner. If this somatic attention mechanism plays some role, it is unlikely to be the whole explanation because it would not explain why contamination-related disgust seems to produce the strongest responses.

Another cultural factor that may influence how people respond with disgust could involve how a society falls along the individualism-collectivism continuum. The majority of the research into disgust and contamination has been in Western societies that are more individualist than collectivist, such as the U.S. and European countries. In their study on emotions in five sub-Saharan African nations, Kim-Prieto and Eid (2004) found that Ghana was among the highest in collectivism. They also found that negative emotions, such as fear and anger, seemed to be evaluated more negatively in African collectivist nations than in Western or Eastern nations high in collectivism. Kim-Prieto and Eid did not include disgust among the negative emotions examined, but responses to disgust are likely to be similarly negative. Members of collectivist societies tend to endorse lower levels of emotional expressions, especially negative emotions (Matsumoto et al., 2008) so it stands to reason that if fear and anger were highly undesirable emotional states in Ghana (Kim-Prieto and Eid, 2004), that disgust would be responded to in kind. Thus, a reluctance to express negative emotions in a collectivist society might lead to an underestimation of self-reported disgust, not an overestimation, supporting a cultural role in the higher levels of disgust in Ghana.

Fincher et al. (2008) and Murray and Schaller (2010) found strong associations between the disease prevalence index and level of collectivism suggesting that collectivism developed in response to historical disease threats. Thus, if collectivism plays a role in shaping the extent of sensitivity to disgust and contamination, the disease prevalence hypothesis would suggest that the history of disease threats might also be shaping the nature of the collectivist trends in a society. Disgust sensitivity has been addressed in two other collectivist countries, but neither country is rated high on disease prevalence (Kuwait: −0.34; South Korea: −0.11; Murray and Schaller, 2010) and neither study made direct comparisons to other Western countries (Al-Fayez et al., 2009; Kang et al., 2012). Disgust needs to be assessed in more countries that vary in historical disease threat and collectivism to clarify these relationships.

### Implications for disgust and contamination sensitivity in African Americans

The results from the present study, with Ghanaians’ scores on the disgust and contamination sensitivity scales higher than a comparable U.S. sample, has implications for recent studies on contamination sensitivity among African Americans. Several studies have found that African Americans show higher levels of disgust (Haidt et al., 1994) and contamination sensitivity (Williams and Turkheimer, 2007; Williams et al., 2012) than European Americans (although Williams et al., 2012, using a different disgust scale concluded that African Americans did not exhibit higher disgust). Disgust and contamination sensitivity tend to be correlated in Western samples (Mancini et al., 2001; Olatunji et al., 2005) and the Ghanaian students showed the same significant correlational relationship between contamination and most of the disgust measures (Table 3). These results suggest a strong trend for Ghanaians and African Americans to both produce scores indicating higher disgust and contamination sensitivities (Williams and Turkheimer, 2007: mean Padua total score for Whites = 7.08, mean Padua total score for Blacks = 11.84; this study: mean Padua total score for Ghanaians = 16.35). Moreover, the Ghanaian results may complicate the explanation that Williams and Turkheimer (2007) proposed to explain high contamination sensitivity in African American samples: cultural shifts to counteract nineteenth and early twentieth century negative stereotypes in regards to cleanliness. Similar such cultural shifts were unlikely to have occurred in Ghana.

Alternatively, it is also possible that the greater contamination concerns of African Americans are not due to cultural shifts in the last century, but represent a deep-seated behavioral legacy carried over from West Africa during the slave trade and continued until today. We do not suggest a genetic legacy, but rather a cultural one reminiscent of the way certain aspects of African culture survived the slavery period and are practiced today by the Gullah people in coastal regions in the southern United States (Pollitzer, 1999). However, it is also possible that heightened sensitivities to disgust and contamination in African Americans comes from a specific adaptation to a history of living in regions of the world, such as West and Central Africa, where the greatest threats of contagious disease can be found (Murray and Schaller, 2010). Then, if African Americans show similar emotional traits evident in the Ghanaians in the current study, these traits may represent biological as well as cultural adaptations to such threats. Further comparative research is needed to elucidate the similarities and differences between West Africans’ and African Americans’ high disgust and contamination sensitivity.

### Universal nature of disgust

Claims that disgust is a universal emotion centered around food and contaminating agents (Curtis and Biran, 2001; Rozin et al., 2008; Oaten et al., 2009) were supported in the current study as Ghanaian undergraduates showed elevated disgust sensitivity levels to the categories that were designed to disgust Western individuals. However, their pattern of responses to the DS-R, as indicated by the factor analysis, showed some differences compared to what is typically found in samples from the U.S. Thus, there may be variation in the types of exemplars that should be used for an African disgust study that were missing using a Western-based scale. While it is assumed that experiencing disgust is universal, the specific foods, animals, hygiene behaviors, etc. that produce disgust often vary with culture and possibly with levels of disease threats. For instance, cockroaches are considered to be a strong elicitor of disgust in the West, and items about cockroaches appear on all disgust surveys. However, the cockroach item on the DS-R garnered the lowest disgust scores for Ghanaians and was one of only two items where the U.S. students reported significantly higher disgust (the other was the item describing the removal of a glass eye). The cockroach item on the TDDS-pathogen was also the only TDDS item that the American students rated significantly more disgusting than the Ghanaian students. This suggests additional research is needed to better understand the nature of disgust elicitors in Ghana.

Besides the level of exposure to contaminating agents, we might expect countries or societies to vary in their tendencies toward disgust based on several other cultural variables, such as cultural values (Rozin et al., 2008), views of religion and purity (Haidt et al., 1997), political orientations (Inbar et al., 2012), and language usage (Schweiger Gallo et al., 2012). For example, Americans have been observed to be higher in disgust sensitivity than Dutch respondents (Olatunji et al., 2006) and Olatunji et al. (2009) found that the three-factor structure of the DS-R was not invariant across four European countries. If country-level differences in disgust can emerge within somewhat similar Western contexts, it is critical to test the nature of disgust in non-Western countries as well to better understand how the variance in cultural factors relates to the nature of disgust and other emotions. The current study sought to expand the coverage of countries previously addressed and to start the process to fully understand the factors that influence differences in disgust sensitivity.

### Limitations

The fact that our Ghanaian sample came from a university population could potentially limit the nature of our conclusions. Undergraduates taking psychology courses at the University of Ghana likely come from among the more affluent and westernized families in Ghana, and therefore we might expect more similarities than differences when comparing them to American students. We also might expect them to hold fewer traditional ideas and customs than more rural-living Ghanaians. However, this study distinctly found differences between the two samples of students across disgust and contamination scales, which should indicate that these differences were strong given the potential for similar lifestyle effects. Working with university populations in both countries could limit the generalizability of our findings to non-student populations, and it would be desirable to measure sensitivity to disgust and contamination in more traditional-living West Africans.

Looking at the over-arching affects of disease prevalence may miss interactions that suggest disease threat is not the only factor generating cultural differences in disgust. In impoverished areas of regions that score high on the disease prevalence index, hand washing with soap after changing a diaper or using a toilet is often rare (e.g., range 0–20% in impoverished areas of Burkino Faso, Ghana, Nigeria, India, and Brazil; Scott et al., 2007) where disease prevalence theory might expect it to be more customary. This variability in washing after contact with contaminating substances suggests unseen dirt or fecal contamination may not be naturally disgust-evoking in all societies. Thus, other factors, such as culture, economics, rural-urban differences, and access to clean water may also be important determinants for what is considered disgusting in various countries. Along these lines, Fincher et al. (2008) and Schaller and Murray (2008) found that indices of economic wealth also correlated with collectivism and extraversion, both of which could influence the way people may respond to contaminating agents. Thus, further work establishing how sensitive rural and impoverished populations are to scenarios and stimuli that evince contamination and disgust is necessary to understand how contextual factors might influence such emotional responses.

Alpha values were generally lower in the Ghanaian sample than in the U.S. sample, except for the religiosity measure. There were two procedural differences in how the students in Ghana and the U.S. took the surveys: Ghanaian students completed paper versions of the surveys in a classroom setting and American students completed online versions of the surveys individually on a computer. We doubt that these procedural differences played a major role in the outcome of the surveys or their reliability estimates. Comparable disgust data from a different survey study (*n* = 118) in one of our labs in Philadelphia, USA, using paper versions of the DS-R, produced almost identical means to the online data (*M* = 2.22, SD = 0.69, for current study; *M* = 2.29, SD = 0.57, for the paper version), even though the alpha values varied (α = 0.91 for online; α = 0.82 for paper version). Thus, it is more likely that the lower alpha values are due to cultural variability in how Ghanaian students internalized the survey items. As mentioned above, the items are Western-oriented and it might be that variation in a factor such as cultural knowledge generated variability in their responses.

Disgust sensitivity was measured in this study with surveys as in most other disgust studies. However, survey data may be limited cross-culturally due to variation in interpreting survey items (e.g., the problem with low alpha for the PVD-GA). We adjusted the items in the disgust scales to represent Ghanaian culture, but directly assessing disgust tendencies with culturally relevant stimuli in a laboratory setting may lead to a better understanding of the nature of disgust in different regions. The addition of measures of daily experiences of disgust or of the display rules that might influence the expression of disgust will aid in understanding how a collectivist society may play a role in altering the nature of how Africans respond emotionally.

## Conclusion

The results of the present study support the claim that people living in an environment in which there has been threatening levels of infectious diseases would be expected to be more sensitive to disgust-evoking situations, especially situations that connote contamination (Schaller, 2011). Ghanaian students were found to score significantly higher than students in the U.S. on the two disgust sensitivity scales (DS-R, TDDS), a contamination sensitivity scale (Padua Inventory), and a scale that measuring avoidance of germs (PVD-GA). That large effect sizes were found for the contamination items on the DS-R, and the contamination sensitivity measures of the Padua and the PVD-GA, also supported the importance of contaminating elements driving cross-national differences in disgust. Furthermore, we found that contamination sensitivity (measured by the Padua) mediated the country differences in disgust sensitivity levels. Taken together, this set of findings fit well with the disease prevalence theory that regions potentially high in infectious rates should be more sensitive to contaminating and disgust-evoking elements.

Even though these surveys were created to address the kinds of domains that Americans were known to find disgusting, Ghanaians also found the survey items disgusting, but to an even greater extent. However, the factor structure of the DS-R did not resemble the standard three factors present in eight different national samples, including a collectivist country such as Japan (Olatunji et al., 2009). Therefore, the nature of disgust sensitivity in Ghana may be fundamentally different from other populations. This difference in the pattern of responses to the DS-R could be due to several factors, including a history of living in a region with the threat of contagious diseases, Ghanaian heightened responses, or some other cultural variable, such as the nature of Ghanaian collectivism or emotional display rules. Comparative studies are needed to examine how emotions, including disgust, differ between non-Western and Western populations and to further elucidate the role of historical or present infectious disease threats on emotional responses.

## Conflict of Interest Statement

The authors declare that the research was conducted in the absence of any commercial or financial relationships that could be construed as a potential conflict of interest.

## References

[B1] Al-Fayez G. Awadalla A. Arikawa H. Templer D. I. Hutton S. (2009). Body elimination attitude family resemblance in Kuwait. Int. J. Psychol. 44, 410–417 10.1080/00207590802644733 22029659

[B2] Ameka F. (2002). Cultural scripting of body parts for emotions: on “jealousy” and related emotions in Ewe. Pragmat. Cogn. 10, 27–58 10.1075/pc.10.12.03ame

[B3] Cohen A. B. Malka A. Rozin P. Cherfas L. (2006). Religion and unforgivable offenses. J. Pers. 74, 85–118 10.1111/j.1467-6494.2005.00370.x 16451227

[B4] Curtis V. Aunger R. Rabie T. (2004). Evidence that disgust evolved to protect from risk of disease. Proc. Biol. Sci. 271, S131–S133 10.1098/rsbl.2003.0144 15252963 PMC1810028

[B5] Curtis V. Biran A. (2001). Dirt, disgust, and disease: is hygiene in our genes? Perspect. Biol. Med. 44, 17–31 10.1353/pbm.2001.0001 11253302

[B6] Davey G. C. L. (1994). Self-reported fears to common indigenous animals in an adult UK population: the role of disgust sensitivity. Br. J. Psychol. 85, 541–554 10.1111/j.2044-8295.1994.tb02540.x 7812671

[B7] Deacon B. Olatunji B. O. (2007). Specificity of disgust sensitivity in the prediction of behavioral avoidance in contamination fear. Behav. Res. Ther. 45, 2110–2120 10.1016/j.brat.2007.03.008 17481576

[B8] Duncan L. A. Schaller M. Park J. H. (2009). Perceived vulnerability to disease: development and validation of a 15-item self-report instrument. Pers. Individ. Diff. 47, 541–546 10.1016/j.paid.2009.05.001

[B9] Dzokoto V. (2010). Different ways of feeling: emotion and somatic awareness in Ghanaians and Euro-Americans. J. Soc. Evol. Cult. Psychol. 4, 68–78

[B10] Dzokoto V. A. Okazaki S. (2006). Happiness in the eye and the heart: somatic referencing in West African emotion lexica. J. Black Psychol. 32, 117–140 10.1177/0095798406286799

[B11] Ekman P. (1992). An argument for basic emotions. Cogn. Emot. 6, 169–200 10.1080/02699939208411068

[B12] Fincher C. L. Thornhill R. Murray D. R. Schaller M. (2008). Pathogen prevalence predicts human cross-cultural variability in individualism/collectivism. Proc. Biol. Sci. 275, 1279–1285 10.1098/rspb.2008.0094 18302996 PMC2602680

[B13] Geurts K. L. (2002). On rocks, walks, and talks in West Africa: cultural categories and ananthropology of the senses. Ethos 30, 178–198 10.1525/eth.2002.30.3.178

[B14] Graham J. Haidt J. (2010). Beyond beliefs: religions bind individuals into moral communities. Pers. Soc. Psychol. Rev. 14, 140–150 10.1177/1088868309353415 20089848

[B15] Haidt J. McCauley C. Rozin P. (1994). Individual differences in sensitivity to disgust: a scale sampling seven domains of disgust elicitors. Pers. Individ. Diff. 16, 701–713 10.1016/0191-8869(94)90212-7

[B16] Haidt J. Rozin P. McCauley C. Imada S. (1997). Body, psyche, and culture: the relationship between disgust and morality. Psychol. Dev. Soc. 9, 107–131 10.1177/097133369700900105

[B17] Inbar Y. Pizarro D. Iyer R. Haidt J. (2012). Disgust sensitivity, political conservatism, and voting. Soc. Psychol. Pers. Sci. 3, 537–544 10.1177/1948550611429024

[B18] Kang J. I. Kim S. J. Cho H. J. Jhung K. Lee S. Y. Lee E. (2012). Psychometric analysis of the Korean version of the Disgust Scale-Revised. Compr. Psychiatry 53, 648–655 10.1016/j.comppsych.2011.06.005 21831367

[B19] Kim-Prieto C. Eid M. (2004). Norms for experiencing emotions in Sub-Saharan Africa. J. Happiness Stud. 5, 241–268 10.1007/s10531-004-8787-2

[B20] Mancini F. Gragnani A. D’Olimpio F. (2001). The connection between disgust and obsessions and compulsions in a non-clinical sample. Pers. Individ. Diff. 31, 1173–1180 10.1016/S0191-8869(00)00215-4

[B21] Matchett G. Davey G. C. L. (1991). A test of the disease avoidance model of animal phobias. Behav. Res. Ther. 29, 91–94 10.1016/S0005-7967(09)80011-9 2012593

[B22] Matsumoto D. Yoo S. H. Fontaine J. Anguas-Wong A. M. Arriola M. Ataca B. (2008). Mapping expressive differences around the world: the relationship between emotional display rules and individualism versus collectivism. J. Cross Cult. Psychol. 39, 55–74 10.1177/0022022108315489

[B23] Murray D. R. Schaller M. (2010). Historical prevalence of disease within 230 geopolitical regions: a tool for investigating origins of culture. J. Cross Cult. Psychol. 41, 99–108 10.1177/0022022109349510

[B24] Oaten M. Stevenson R. J. Case T. I. (2009). Disgust as a disease-avoidance mechanism. Psychol. Bull. 135, 303–321 10.1037/a0014823 19254082

[B25] Olatunji B. O. Adams T. Ciesielski B. David B. Sarawgi S. Broman-Fulks J. (2012). The three domains of disgust scale: factor structure, psychometric properties, and conceptual limitations. Assessment 19, 205–225 10.1177/1073191111415368 22218974

[B26] Olatunji B. O. Moretz M. W. McKay D. Björklund F. de Jong P. J. Haidt J. (2009). Confirming the three-factor structure of the Disgust Scale-Revised in eight countries. J. Cross Cult. Psychol. 40, 234–255 10.1177/0022022108328918

[B27] Olatunji B. O. Sawchuk C. N. de Jong P. J. Lohr J. M. (2006). The structural relation between disgust sensitivity and blood–injection–injury fears: a cross-cultural comparison of US and Dutch data. J. Behav. Ther. Exp. Psychiatry 37, 16–29 10.1016/j.jbtep.2005.09.002 16274661

[B28] Olatunji B. O. Sawchuk C. N. Lohr J. M. de Jong P. J. (2004). Disgust domains in the prediction of contamination fear. Behav. Res. Ther. 42, 93–104 10.1016/S0005-7967(03)00102-5 14744526

[B29] Olatunji B. O. Williams N. L. Tolin D. F. Abramowitz J. S. Sawchuk C. N. Lohr J. M. (2007). The disgust scale: item analysis, factor structure, and suggestions for refinement. Psychol. Assess. 19, 281–297 10.1037/1040-3590.19.3.281 17845120

[B30] Olatunji B. O. Sawchuk C. N. Arrindell W. A. Lohr J. M. (2005). Disgust sensitivity as a mediator of the sex differences in contamination fears. Pers. Individ. Diff. 38, 713–722 10.1016/j.paid.2004.06.012

[B31] Pollitzer W. S. (1999). The Gullah People and Their African Heritage. Athens, GA: University of Georgia Press

[B32] Rozin P. Fallon A. (1987). A perspective on disgust. Psychol. Rev. 94, 23–41 10.1037/0033-295X.94.1.23 3823304

[B33] Rozin P. Haidt J. McCauley C. R. (2008). “Disgust,” in Handbook of Emotions, 3rd Edn, eds Lewis M. Haviland-Jones J. M. Feldman Barrett L. (New York: Guilford), 757–776

[B34] Sanavio E. (1988). Obsessions and compulsions: the Padua Inventory. Behav. Res. Ther. 26, 169–177 10.1016/0005-7967(88)90116-7 3365207

[B35] Sawchuk C. N. Lohr J. M. Tolin D. F. Lee T. C. Kleinknecht R. A. (2000). Disgust sensitivity and contaminationfears in spider and blood–injection–injury phobias. Behav. Res. Ther. 38, 753–762 10.1016/S0005-7967(99)00093-5 10937424

[B36] Schaller M. (2011). The behavioural immune system and the psychology of human sociality. Philos. Trans. R. Soc. Lond. B Biol. Sci. 366, 3418–3426 10.1098/rstb.2011.0029 22042918 PMC3189350

[B37] Schaller M. Duncan L. A. (2007). “The behavioral immune system: its evolution and social psychological implications,” in Evolution and the Social Mind: Evolutionary Psychology and Social Cognition, eds Forgas J. P. Haselton M. G. von Hippel W. (New York: Psychology Press), 293–307

[B38] Schaller M. Murray D. R. (2008). Pathogens, personality, and culture: disease prevalence predicts worldwide variability in sociosexuality, extraversion, and openness to experience. J. Pers. Soc. Psychol. 95, 212–221 10.1037/0022-3514.95.1.212 18605861

[B39] Scherer K. R. (1997a). Culture effects on emotion-antecedent appraisal. J. Pers. Soc. Psychol. 73, 902–922 10.1037/0022-3514.73.5.902

[B40] Scherer K. R. (1997b). Profiles of emotion antecedent appraisal: testing theoretical predictions across cultures. Cogn. Emot. 11, 113–150 10.1080/026999397379962

[B41] Schienle A. Stark R. Walter B. Vaitl D. (2003). The connection between disgust sensitivity and blood-related fears, faintnesssymptoms, and obsessive–compulsiveness in a non-clinical sample. Anxiety Stress Coping 16, 185–193 10.1080/10615806.2003.10382972

[B42] Schweiger Gallo I. Fernández-Dols J. M. Pablo-Lerchundi I. Gollwitzer P. M. (2012). “Analyzing the Spanish (“asco”) and German (“Ekel”) equivalents of disgust from a prototype perspective,” in Poster Presented at Association for Psychological Science Annual Conference, Chicago, IL

[B43] Scott B. Curtis V. Rabie T. Garbrah-Aidoo N. (2007). Health in our hands, but not in our heads: understanding hygiene motivation in Ghana. Health Policy Plan. 22, 225–233 10.1093/heapol/czm014 17526639

[B44] Tybur J. M. Lieberman D. Griskevicius V. (2009). Microbes, mating, and morality: individual differences in three functional domains of disgust. J. Pers. Soc. Psychol. 97, 103–122 10.1037/a0015474 19586243

[B45] Williams M. T. Abramowitz J. S. Olatunji B. O. (2012). The relationship between contamination cognitions, anxiety, and disgust in two ethnic groups. J. Behav. Ther. Exp. Psychiatry 43, 632–637 10.1016/j.jbtep.2011.09.003 21946040

[B46] Williams M. T. Turkheimer E. (2007). Identification and explanation of racial differences on contamination measures. Behav. Res. Ther. 45, 3041–3050 10.1016/j.brat.2007.08.013 17935695 PMC2211632

